# Co-existence of sarcoidosis and pulmonary embolism

**DOI:** 10.5578/tt.202402917

**Published:** 2024-06-11

**Authors:** Övgü VELİOĞLU YAKUT, Miraç ÖZ, Serhat EROL, Öznur YILDIZ, Özlem ÖZDEMİR KUMBASAR

**Affiliations:** 1 Department of Pulmonary Diseases, Ankara University Faculty of Medicine, Ankara, Türkiye

Dear Editor,

Sarcoidosis is a multisystem, granulomatous disease of unknown cause (1). Venous thromboembolism (VTE), including deep vein thrombosis (DVT) and pulmonary embolism (PE), is a common condition associated with high morbidity and mortality. VTE risk factors include immobilization, hospitalization, surgery, malignancy, aging, thrombophilic status, and the use of certain drugs (2). Recently, chronic inflammation has been recognized as an inde- pendent risk factor for VTE. Chronic activation of coagulation by inflammatory cytokines is believed to be the key pathogenesis behind this association. Epidemiological studies have shown an increased incidence of VTE in a variety of chronic immune-mediated inflammatory disorders such as rheumatoid arthritis, vasculitis, and inflammatory intestinal disease (3). Few previous retrospective and observational studies have suggested an increased risk of venous thromboembolic disease in patients with sarcoidosis. Patients with sarcoidosis have been found to have an increased risk of pulmonary embolism compared to the normal population. Although the exact mechanism is not clear, it suggests that sarcoidosis, like other chronic inflammatory diseases, may lead to hypercoagulability with chronic inflammation (4,5). Macrophages and activated leukocytes increase thrombin activation and fibrin formation in sarcoidosis. There is not enough evidence on the incidence and source of hypercoagulability and hypofibrinolysis in sarcoidosis. The true incidence of concomitant sarcoidosis and VTE is unknown. There are some complications associated with longterm use of corticosteroids for the treatment of sarcoidosis such as presence of vascular granuloma, compression of the pulmonary arteries due to mediastinal or hilar lymph nodes, bidirectional inflammation and coagulation process. It is suggested that presence of comorbidities are responsible for the association of sarcoidosis Sarcoidosis and pulmonary embolism and VTE (6). In a study by Swigris et al. analyzing the death records from 1988 to 2017, the authors found that patients with the sarcoidosis were more than twice the risk of developing PE compared to the general population (7). A study at the Mayo Clinic showed significant increase statistically at the risk of VTE in patients with sarcoidosis in a systematic review of observational studies (8). Patompong et al. compared 345 sarcoidosis cases and 345 control groups from 1976 to 2013 in terms of DVT and PE and observed an increased risk of VTE among patients with sarcoidosis (4). Yaqoob et al. repeated this study with a larger number of patients and found that sarcoidosis was associated with an increased risk of VTE (5). We present a case of PE with no underlying risk factors other than sarcoidosis.

**Figure 1:**
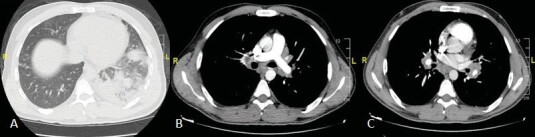
Thorax CT (A) Nodular consolidation and ground glass areas in the lower lobe of the left lung (B-C) Lymphadenopathies in the subcarinal, bilateral hilar, right upper paratracheal, and precarinal areas.

**Figure 2:**
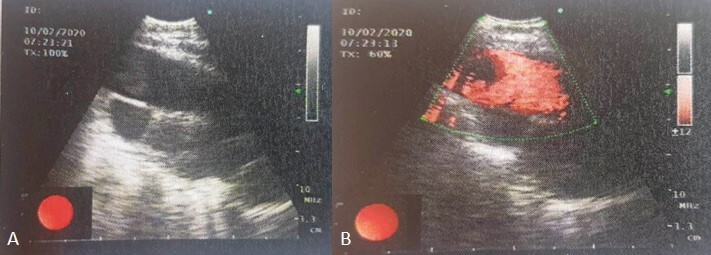
EBUS images (A) Thrombus in the pulmonary artery (B) Blood flow in the pulmonary artery.

**Figure 3:**
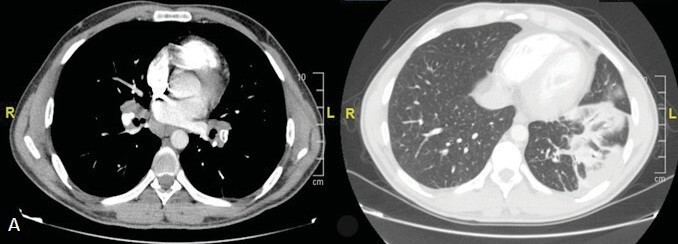
Pulmonary CT angiography (A) Thromboembolism in the lobar and segmental branches of lower lobe of the left lung (B) Consolidation area compatible with infarct in the left lower lobe.

Our case was a 32-year-old male patient presented with chest pain and hemoptysis that started a week ago. He did not have any known disease. In the left lung, infiltration was observed in the posteroanterior chest X-ray and levofloxacin treatment had been started empirically in another center. On thorax computed tomography (CT), bilateral hilar, paratracheal, precarinal, subcarinal lymphadenopathies, nodular consolidation and ground glass areas in the left lung lower lobe, the largest of which is approximately 2 cm in diameter, without air bronchogram, pleural effusion on the left lung were detected (Figure 1). The patient was hospitalized in our clinic for further examination with the preliminary diagnoses of pneumonia, PE, tuberculosis, sarcoidosis, and lymphoproliferative disease. On physical examination, his vital signs were stable, and crackles were heard under the scapula on the left hemithorax on auscultation. Some biochemical measurements were high such as aspartate amino transaminase 89 U/L, alanine amino transaminase 103 U/L, C-reactive protein 80 mg/L, d-dimer 634 ng/dL. Since there were diffuse mediastinal lymph nodes, positron emission tomography (PET-CT) was performed to exclude lymphoproliferative diseases, and F-18 fluoro2-deoxy-glucose (FDG) uptake was found to be 8.9 in mediastinal lymph nodes. Transbronchial needle aspiration biopsy (TBNA) was taken using endobronchial ultrasonography (EBUS) from the lymph nodes 11R, 10R, 4R, 7, 11L and 4L. During EBUS ultrasonography, images showed an appearance that may be compatible with thrombus in the pulmonary artery (Figure 2). Pulmonary CT angiography was performed because of that. Thromboembolism was observed in the lobar and segmental branches of the lower lobe of the left lung, and a consolidation area was seen in the left lower lobe, which may be compatible with infarct (Figure 3). Low molecular weight heparin (enoxaparin) was started 0.1 mg/kg twice a day. Mean pulmonary artery pressure was measured as 30 mmHg on transthoracic echocardiography, and right ventricular size and movements were found to be normal. Bilateral lower extremity venous doppler ultrasonography performed for etiology and no thrombus was detected. Nonnecrotizing granulomatous lymphadenitis was determined on cytological examination of TBNA samples. Serum ACE level and urinary calcium levels were found high, 104.5 U/L, 102 mg/24 hours, respectively. No uveitis was detected in the eye examination. Rheumatological disease was excluded by the rheumatology department. PPD was anergic. The patient was accepted as sarcoidosis. Pulmonary function tests were found to be normal. He was followed up with oral anticoagulant therapy for PE. While there was no treatment indication for sarcoidosis, the patient was followed up without treatment.

It is reasonable that sarcoidosis may cause inflammation and hypercoagulability that begins before the presence of clinically evidence of disease. Recent studies in patients with sarcoidosis suggest that these patients have greater risk for VTE than the healthy population. If there is clinical suspicion of VTE in patients with sarcoidosis, further investigations should be performed. More research is needed to clarify the relationship between sarcoidosis and VTE.
